# Occupational Health Nurses’ Perceptions in Work Ability Risk Management and Analysis

**DOI:** 10.1007/s10926-025-10282-7

**Published:** 2025-03-15

**Authors:** Johanna Sirkka, Riitta Suhonen, Juha Liira, Minna Stolt

**Affiliations:** 1 Department of Nursing Science, University of Turku, Turku, Finland; 2 Department of Nursing Science and Director of Nursing, University of Turku, Turku University Hospital, the Wellbeing Services County of Southwest Finland, Turku, Finland; 3 Clinical Department, University of Turku, Turku, Finland; 4 University of Turku, Department of Nursing Science, Turku, Finland, and the Wellbeing Services County of Satakunta, Pori, Finland

**Keywords:** Occupational health nurse, Thematic interview, Work ability risk management, Work ability risk analysis

## Abstract

**Purpose:**

Occupational health nurses (OHN) play a key role in identifying and managing work ability risks, as they have close interaction with employees and the customer organization, and they monitor work ability in multiple ways. The study aimed to describe OHNs’ perceptions of work ability risk management and analysis (WARMA) and identify promoting and hindering factors.

**Methods:**

A descriptive qualitative study with semi-structured thematic interviews was conducted in May–June 2023, using purposive sampling of ten OHNs. The data were analyzed using both inductive and deductive approaches.

**Findings:**

OHNs perceived management and analysis of work ability risks as important work. The management and analysis of work ability risks was described as the central core work of occupational health care, which is carried out at the level of the customer organization and at the individual level. Factors promoting the management and analysis of work ability risks are electronic tools, time resources, occupational health cooperation, multi-professional cooperation, and personal experience. Factors hindering WARMA are insufficient time resources and productivity pressures.

**Conclusion:**

OHNs’ perceptions of WARMA varied. There are multiple factors that promote or hinder WARMA which require consideration at individual and organizational levels. The findings of this study provide a basis for further research that could focus on measuring OHNs' overall competence in WARMA.

## Introduction

Managing and analyzing work ability risks is the core of an occupational health nurses (OHN) work, with the aim of promoting and maintaining work ability and preventing work disability. Work ability risk management and analysis (WARMA) is a cross-cutting continuous process bringing together the key functions of work ability management (promotion), early response (primary prevention), sickness absence management with sick leave follow-up (secondary prevention), and return-to-work support (tertiary prevention), implemented by occupational health services [1–3]. Preventing work disability is a key political and social goal in many societies. This goal is emphasized as working life is continuously changing, and so are the threats of disability [4–6]. For example, sick leave and disability due to psychosocial burden factors have increased [4]. In addition, the age of the working population is increasing. Occupational health care should be able to respond to these changes in cooperation with stakeholders. Work disability has significant negative health, economic, and social effects at the level of both the individual and society [4, 7, 8]. International organizations, such as the World Health Organization [9], International Labour Organization [10], and International Commission on Occupational Health [11], play a key role in shaping global occupational health policy, which influences the assessment and monitoring of working conditions and, consequently, the expertise required in occupational health care. Occupational health services, advising employers on improving working conditions and monitoring workers' health, do not cover most of the working population worldwide [12]. For example, in Finland, statutory occupational health care covers more than 90% of employees, meaning almost two million people of working age [13]. In 2023, there were slightly more than 1000 members on the Finnish OHN Association's membership register [14]. Given the high number of employees under the occupational health services, OHNs have an important role in identifying, managing, and analyzing work ability risks, and it is essential they have the competence for this. In Finland, the qualification of an OHN includes occupational health care specialization studies in addition to the training of a registered health nurse [15–17]. Finland is involved in Federation of Occupational Health Nurses within the European Union as a national representative [18]. According to the organization, Occupational Health Nursing aims at securing the health, safety, and well-being of the workforce. This is achieved through assessing, monitoring, and promoting the health status of the workers, and developing strategies to improve the working conditions and the total environment. [19]. OHNs are required to have competence, meaning knowledge and skills, qualifications, and the ability to apply their knowledge to changing practical situations and customer cases on different levels, such as primary prevention, health assessment, sickness absence management, and rehabilitation. [20–23]. In their work, OHNs need extensive qualifications, including observation, inspection, assessment, research, and health promotion; compliance with confidentiality and ethical rules; and collaboration with the team [23–25]. In addition, experiences and attitudes are related to competence [26] as professional competence can comprehensively be defined as a set of knowledge, skills, attitudes, and performance [27, 28]. Due to extensive competence requirements, OHNs need opportunities to develop and maintain their professional competence [23, 29].

Previous research on the management and analysis of work ability risks is scarce and fragmented. Occupational health care physicians perceive management and analysis of work ability risks as important when a confidential relationship between physician and customer, teamwork, and knowledge about the workplace are central [30]. Identifying work ability risks is a key part of the work of occupational health professionals. For example, common main areas for OHNs working in the United Kingdom have been described as assessment and management of work-related health hazards, assessment of disability and fitness for work, health promotion, and assessment of workforce health promotion needs [22]. Furthermore, identification of impaired work ability is important in the work of an OHN [31]. Employees working in occupational health care support work ability at customer organization and individual levels [32]. Identification and assessment of the risks of workplace hazards, management of occupational hazards, and work-related diseases are key areas in occupational health care [33]. Occupational health care professionals’ competence to manage and analyze work ability risks has been reported to be partly at a good level, but deficits in central areas have also been identified [1]. Given its importance, WARMA research on OHNs’ attitudes and experiences in the management and analysis of work ability risks seems to be lacking.

## Objectives

The aim of this study was to describe OHNs’ perceptions of WARMA and identify promoting and hindering factors. Emphasis was placed on identifying OHNs' attitudes and experiences in managing and analyzing work ability risks, as OHNs play an important role in identifying and developing key areas in supporting work ability and promoting health. The information can be used to improve work ability risk management processes in occupational health care.

The specific research questions were as follows:

1. What are OHNs’ attitudes to managing and analyzing work ability risks?
2. What experiences do OHNs have in managing and analyzing work ability risks?
3. Which factors promote OHNs’ management and analysis of work ability risks?
4. Which factors hinder OHNs’ management and analysis of work ability risks?
5. How do OHNs manage and analyze work ability risks?

## Methods

### Design, Data Collection, and Participants

A descriptive qualitative study was conducted. Purposive sampling [34] was used to recruit OHNs from public, private, and integrated occupational health services in Finland by placing an invitation to participate in the study in the professional journal of the Finnish OHNs’ association. The participant inclusion criteria were: 1) OHN; 2) works in public, private, or integrated occupational health care; and 3) the participant has experience in managing and analyzing work ability risks.

The data were collected through semi-structured individual thematic interviews between January and May 2023. An interview guide [34] based on previous research [21, 35, 36] and the current legislation [16] was constructed. The interview guide had two parts: a theme interview and a customer case. The first part, the theme interview, focused on factors promoting and hindering the management and analysis of work ability risks, identifying the management and analysis of work ability risks, treatment, and rehabilitation measures. The questions were related to perceptions and, in particular, attitudes and experiences of OHNs regarding the management and analysis of work ability risks.

The second part of the interview form consisted of handling an imaginary customer case related to the management and analysis of work ability risks. In this part, the OHNs explained how they would practically manage and analyze work ability risks in different situations. The customer case dealt with the work ability and work ability challenges of a nurse who returned to work from maternity leave. OHNs described how they would support a person in different situations related to supporting work ability. Through the customer case, more detailed information was available about OHNs' competence and functioning in different situations, as well as how they apply their competence in the management and analysis of work ability risks [37, 38].

As background information, individual and the organization-related information was collected: age, highest professional education level, working in public, private, or integrated occupational health care and work experience in the field of occupational health care.

The interviews lasted 60–90 min each. The recorded interviews were transcribed verbatim. After that, the researchers familiarized themselves with the material by reading it several times, which helped in understanding the overall picture.

### Data Analysis

The data were analyzed using both inductive and deductive approaches. Frequency and percentage distributions, as well as average and range, were used to describe the background data. The analysis of the first part of the interview material was conducted using data-driven, inductive content analysis, progressing from specific observations to general conclusions. [39]. First, guided by the research questions, corresponding units of meaning were identified, such as words, sentences, or paragraphs, which were extracted from the data into a table. These units of meaning were marked and then the expressions were condensed and coded in such a way that the essential content remained. A total of 202 codes were accumulated. Reductions with similar meanings were combined into subcategories according to their content, and the subcategories were given a conceptual name covering the content. The analysis was continued by assembling the subcategories into broader entities, from which the super-categories were formed by abstracting. The categories were named based on the reductions they contain, to answer the research questions (Table 1).

**Table 1:** An example of the progress of the first part of the interview form data analysis: Factors promoting the management and analysis of work ability risks

Original expression	Simplified codes	Subcategory	Main category
“Above all, that sickness absences are recorded somewhere in the system. In addition, that our system is functional.”	Registration of sickness absences in well-functioning electronic systems	Well-functioning electronic tools	Electronic tools
“That there would be enough time for that. Because if it is not there, you will not be able to implement 100% of it.”	Enough time is needed to be able to implement well	There should be enough time	Time resources
“The fact that you get information shared. Moreover, employers are different. Yes, the awakening of the employer is one thing that contributes. In addition, the employer's motivation and attitude towards those things that it wants, indicate that the employee is also an asset to the organization.”	The employer's perceptions and actions	Well-functioning occupational health cooperation with the customer organization	Occupational health cooperation
“I am grateful in the sense that I have also had the entire occupational health team that has been committed to that, to that work on that side.”	The operation and commitment of the occupational health team	Well-functioning multi-professional collaboration with the occupational health team	Multi-professional cooperation
“Experience helps in this matter, as well. When you have been in different situations and have seen what is good or what works.”	Experience of different situations and what is good or what works	Experience of different situations	Experience

The analysis of the second part of the interview material was conducted using deductive, framework-based content analysis, progressing from general concepts to specific observations [40, 41]. The deductive frame was constructed based on the four sub-areas of WARMA (work ability monitoring, early response, sickness absence, and return-to-work support) [1]. These four sub-areas were used as the upper categories of the analysis matrix.

Expressions that described how the OHNs would act in customer care were extracted. Reduced expressions were formed from similar expressions, which were combined into subcategories. The subcategories were examined using the themes of the analysis matrix and placed under each category. The existing categories guided the creation of subcategories throughout the analysis (Table 2).

**Table 2:** An example of the progress of the second part interview form data analysis

Category	Subcategory	Compound expressions	Original expressions
Work ability monitoring	Promotion	Proactive work ability support	“I would talk about their own resources, that coping, that life change. Another thing is whether it is the phase of returning to full-time work or whether it is something else. After all, they can go over that in a conversation with their supervisor. That their life situation is such that they need to return to full-time work or working hours… That there is a possibility to consider the family situation in working hours. If it seems that there is an uncertain situation with coping, then I would agree to check whether they feel that they would benefit from coming again, then we would see how it goes.”
Early response	Primary prevention	Early support conversation	“I would say that the supervisor should have discussed this matter with the customer. I would either direct them to use the early support model if they have one, and if they do not, I would refer to our occupational health service's instructions on how to act in these situations.”
Sickness absence monitoring	Secondary prevention	Absence alarms	“I would call the customer and tell them that sickness absences are being monitored. We have absence alarms. I would say that I have noticed that they are on a long sick leave, what is going on, and when will the sick leave end. I would check if they had an appointment with an occupational health physician.”
Return-to-work support	Tertiary prevention	A tripartite negotiation and follow-up support	“At this point, I would organize a tripartite negotiation.”

To establish different OHNs’ profiles in management and analysis of work ability risks a four-field analysis model was used. For the analysis, description of uniform modes of operation were combined and four-field analysis using two-dimensional coordinate system was created. [42, 43]. The participants were classified according to whether they knew how to manage and analyze work ability risks in the example customer case well or poorly, and whether they do it a little or a lot in their work.

The entire analysis process was conducted by the first author, supported by the three experienced researchers, who evaluated the analysis and suggested changes when needed. The results were confirmed within the research team.

## Results

### Participants

All participants (n = 10) were women with an average age of 49.5 years, mostly with a degree from the University of Applied Sciences. The majority (70%) worked in private occupational health care. The participants had an average of 18.7 years of work experience in occupational health care services (Table 3).

**Table 3:** Participants’ (n = 10) background information

Background variables	*f*	*%*	*Mean*	*Range*
Age			49.5	34–61
Gender				
Female	10	100		
Educational background				
College	1	10		
University of Applied Sciences	7	70		
University	2	20		
Currently working				
Public occupational health care	1	10		
Private occupational health care	7	70		
Integrated occupational health care	2	20		
Working experience in OHC, years			18.7	4–36

*n* number of participants, *f* frequency, *%* percentage of total

### Attitudes of Occupational Health Nurse Regarding the Management and Analysis of Work Ability Risks

OHNs perceived the management and analysis of work ability risks as *important and useful* (Table 4). The activity was described as cost-effective work, which some of the participants would have liked to invest more in, if there had been enough time available for it. The participants considered that the management and analysis of work ability risks is effective. In addition, experience in managing and analyzing work ability risks was seen as contributing factor to understanding the importance of this work.

**Table 4:** Summarizing table of the main results of the study

Category	Main results
Attitudes of OHNs regarding the management and analysis of work ability risks	Important and useful work: • Cost-effectiveness • Effectiveness • Work in which more time should be invested
OHNs’ experiences of managing and analyzing work ability risks	Central core work of occupational health care: • Cornerstone of occupational health care • The way to understand the root causes behind the factors that threaten the ability to work • Uncertainty Individual-level and organizational-level WARMA: • Knowing and interpreting both levels helps in managing and analyzing work ability risks
Factors promoting the management and analysis of work ability risks	Electronic tools: • Regular use • Timeliness • Correct allocation of measures • Ease of use and reliability Time resources: • Allocating time in advance to the calendar Occupational health cooperation: • Sped up and streamlined processes Multi-professional cooperation: • Regular meetings • Jointly agreed practices • Support and help from colleagues Experience: • Certainty accrues • Competence increases • The work goes smoothly
Factors hindering the management and analysis of work ability risks	Insufficient time resources: • Haste • Many customer organizations Productivity pressures: • Performance targets • Unclear billing processes • Lack of supplementary training • Lack of process descriptions

*OHNs* Occupational health nurses, *WARMA* work ability risk management and analysis

*“It is important. This is very much our most basic task, that we manage and support these things before they become too big problems.”* (Participant 6).

*“This is exactly what is cost-effective and generates savings for the customer organization.” (*Participant 2).

*“The more I do it, the better I understand it and see its meaning.* (Participant 7).

### OHNs' Experiences of Managing and Analyzing Work Ability Risks

The experiences of managing and analyzing work ability risks varied (Table 4). Participants' experiences related to the management and analysis of work ability risks included two main categories: *central core work of occupational health care* and *individual-level and organizational-level WARMA.*

The participants described the management and analysis of work ability risks as the *most central core work of occupational health care*. The management and analysis of work ability risks was perceived as the cornerstone of occupational health care and the starting point, the importance of which is essential for OHNs to realize. Furthermore, OHNs' experiences of managing and analyzing work ability risks were related to the fact that managing and analyzing work ability risks is a way to understand and get to grips with and influence the underlying root causes. Once the root causes have been addressed, health problems affecting work ability can be supported in such a way that the working career continues.

*“It is the core task of occupational health care. It is a means of maintaining and supporting the work ability of employees.” (Participant 1).*

*“It is the cornerstone of the entire occupational health service. That is what our work is based on for the most part.” (Participant 5).*

The participants described experiencing uncertainty in managing and analyzing work ability risks. The reasons given were the lack of clear action plans, fear of exceeding one's own competence, inexperience, and fear of the customer organization or employee's reaction upon contact.

*“That finding the same language with the people with whom we cooperate and trying to manage that situation is sometimes challenging, and this is where the uncertainty comes from.” (Participant 4).*

The OHNs differentiated *between individual-level and organizational-level WARMA*. The participants described how the OHN could benefit from the awareness of the situation by the entire customer organization and mirror the work ability of individuals in relation to the organization's situation, to manage and analyze work ability risks. Knowing and interpreting both levels helps in managing and analyzing work ability risks, as they can often affect each other. For example, smooth work ability management and occupational health cooperation support work ability at the work community level.

*“I see it as not only tracking sickness absence, but also a lot of work that is done before, so that a person remains able to work. That means cooperation: activities at the customer organization level, but also activities at the individual level.” (Participant 10).*

### Factors Promoting the Management and Analysis of Work Ability Risks

Factors promoting the management and analysis of work ability risks consisted of five main categories: *electronic tools, time resources, occupational health cooperation, multi-professional cooperation, and experience* (Table 4)*.*

Electronic tools promoted the management and analysis of work ability risks. With the help of electronic tools, this was implemented in practice, for example, once a week by analyzing the customer organization's sickness absence statistics and selecting and contacting people from there who, in accordance with the practice agreed with the client organization, have received, for example, an electronic absence notification. Another used warning sign of a possible decline in work ability was the high utilization rate of occupational health care. Electronic tools were named as an important issue for enabling the timeliness of the management and analysis of work ability risks and for choosing the right dimensioning of the measures. The participants believed that without electronic tools, the management and analysis of work ability risks has the possibility of being late or measures can be poorly targeted. Therefore, with the help of such tools, it can be ensured that resources are allocated efficiently. In addition, the ease of use and reliability of electronic tools were felt to promote the management and analysis of work ability risks.

*“The development of electronic systems has made it easier to see people at risk through alarms. Those systems help in management, in that you can follow them.” (Participant 7).*

Time resources promoted the management and analysis of work ability risks. The participants perceived it as being worthwhile to regularly mark time in the calendar in advance for managing and analyzing work ability risks, so that the task does not get lost under other work. Many participants set aside time for managing and analyzing work ability risks for larger customer organizations more regularly than for smaller ones.

*“I personally schedule time for managing and analyzing work ability risks in advance.” (Participant 7).*

Well-functioning occupational health cooperation was considered the most important factor in promoting the management and analysis of work ability risks. The size of the customer organization influenced the cooperation, as larger customer organizations usually have better health literacy, resources, motivation, and expertise in occupational health cooperation. A well-functioning occupational health cooperation was realized with the help of jointly agreed operating models and the partner organization's contact persons. In long-term customer relationships, cooperation was refined on a practical level into a unified practice in various work ability processes. Well-functioning occupational health cooperation speeds up and streamlines processes, which in turn eases the employee's situation and brings cost savings.

*“The fact that the customer organization knows what the risk of work ability and its loss mean. And they know how to handle these things smoothly in cooperation with occupational health care. With occupational health cooperation, the best and cheapest solutions are found." (Participant 5).*

Multi-professional cooperation in occupational health care was also perceived as a promoting factor. The occupational health care team's regular meetings and jointly agreed practices were supporting operations, promoting the flow of information, and strengthening professional self-confidence. Discussions in a multi-professional team were important for increasing one's own resources, as it is important to reflect on one's own thoughts in difficult work ability cases.

*"We have a really good cooperation in the occupational health team. I get the necessary support here at my workplace." (Participant 2).*

*“We meet regularly in the customer responsibility team. We discuss difficult cases together, and then there is a common view of what is recommended.” (Participant 1).*

Experience of WARMA was also a promoting factor. The more WARMA was done, the easier it was perceived to be. Experience brings certainty, and as experience accumulates, competence increases. Some of the participants described how experience also helps in choosing an approach in individual work ability cases and in cooperation with customer organizations.

*“I would say that the most important thing is your own experience in managing and analyzing work ability risks." (Participant 6).*

### Factors Hindering the Management and Analysis of Work Ability Risks

Factors hindering the management and analysis of work ability risks consisted of two main categories: *insufficient time resources* and *productivity pressures* (Table 4).

Insufficient time resources, such as a hectic workload, were considered to hinder the management and analysis of work ability risks. The participants invariably stated that they are often busy at work. Due to the rush, the work often should be prioritized, and this can lead to exclusion of the management and analysis of work ability risks. Finding enough time to manage and analyze work ability risks was a concern. Especially at the customer organization level, there is often too little time available for reviewing and analyzing various statistics. This, in turn, can lead to a clouding of the overall picture of the customer organization's work ability situation. The danger that the participants described in this case is that the focus is on the wrong things. Furthermore, participants described how having several customer organizations increases the rush and makes it difficult to manage and analyze work ability risks. Managing large customer organizations was perceived as easier than small ones. Factors affecting this were described as the fact that large customer organizations often invest in workforce management, human resources have prepared process descriptions and offer education to supervisors in this regard, and large customer organizations often recognize the importance of managing and analyzing work ability risks.

*“Managing and analyzing risks isn’t always easy because the work is often busy. I have an extensive customer portfolio and therefore work should be prioritized." (Participant 4).*

*“The lack of time is the most challenging thing here.” (Participant 8).*

Productivity pressures were also perceived as preventing the management and analysis of work ability risks. The descriptions of the participants showed that OHNs balance the amount of billable work. On the one hand, employers of OHNs often set a monthly performance target; on the other hand, they can be expected to bill the customer organization according to actual time use. Invoicing for the management and analysis of work ability risks can be confusing because the work to be done is not necessarily concrete and therefore it can be challenging to justify it. The participants underlined that the invoicing of the management and analysis of work ability risks should be more clearly agreed with the customer organizations. In addition, training and process description would support and facilitate the work.

*“We have performance targets on a monthly level, which come from our own employer. I find this to be a burdensome thing.” (Participant 9).*

*“Training has been quite minimal, but I think that there should be such a basic package, and it should be updated to see what's new. I feel that support is needed.” (Participant 4).*

### OHNs' Profiles in Management and Analysis of Work Ability Risks

**Fig. 1:**
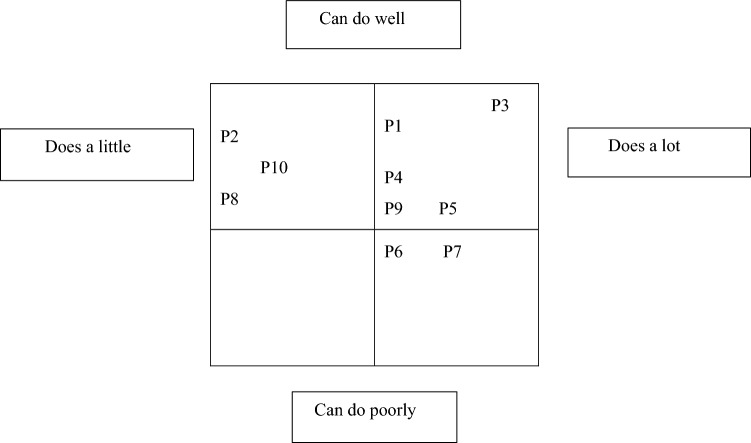
Identified OHN profiles in the management and analysis of work ability risks according to ability and frequency of implementation

Based on the four-field analysis, different OHN profiles were identified based on their perceived ability and frequency of implementation of WARMA (Fig. 1). The majority of OHNs knew well and performed WARMA a lot in their work. Some OHNs considered they had adequate knowledge of WARMA but implemented it rarely.

## Discussion

This study provided novel findings from the perspective of OHNs by examining attitudes, experiences, and factors that promote and hinder the management and analysis of work ability risks. The results of the study emphasize the view that OHNs find the management and analysis of work ability risks to be core work of occupational health care. In addition, OHNs' attitudes to the management and analysis of work ability risks were that the management and analysis of work ability risks is important and effective. The management and analysis of work ability risks was also perceived to be cost-effective. According to the experiences of OHNs, knowing the overall situation of the customer organization is key in managing and analyzing work ability risks. This was felt to help in the management and analysis of both individual-level and customer organization-level work ability risks. Employees working in occupational health care have described how they support work ability both at the customer organization level and at the individual level in previous research, as well [30, 32]. OHNs carry out the management and analysis of work ability risks within the process, as well as guidance and counseling, targeting both the employee and the supervisor as well as, more generally, the customer organization [33]. The results also revealed varying experiences of managing and analyzing work ability risks, where uncertainty is experienced. If the roles, task descriptions, and operational control plans related [6] to the management and analysis of work ability risks were not clear, uncertainty was felt. In addition, inexperience seemed to increase uncertainty.

Managing and analyzing work ability risks is part of the extensive and demanding field of work of an OHN [24], and several factors promoting the management and analysis of work ability risks were found. Well-functioning electronic tools and adequate investments in time promote WARMA. This requires data management skills [24]. Further, cooperation was identified as a factor promoting the management and analysis of work ability risks both in multi-professional activities and in customer organization cooperation. Cooperation has also been found to be meaningful and useful in previous research [6, 25, 30].

Insufficient time resources and a large workload were perceived as factors hindering the management and analysis of work ability risks. These results are in line with those of Nissinen et al. [31]. The situation could be improved by securing sufficient resources and by allocating working time specifically to the management and analysis of work ability risks.

The OHNs suggested in the interviews that they proceed in the same way in customer cases. The management and analysis of work ability risks is often done as part of the overall work of an OHN, and self-assessed competence is considered sufficient. The work would be supported by a process description. Overall, this study provided support for the deductive framework of WARMA (work ability monitoring, early response, sickness absence, and return-to-work support).

### Research Ethics

The study followed the European Union's General Data Protection Regulation (GDPR) 2016/679 [44], and European research ethics practices and guidelines [45]. The study has received ethical approval from the University of Turku Ethics Review Committee (Ethical committee code: 22/2022) and a research permit from the Finnish Association of OHNs. In this study, participation in the research was voluntary, and the subjects were treated respectfully, maintaining trust without violating their human dignity or privacy. The participants received information about the purpose of the study, confidentiality, anonymity, possible risks and benefits, the possibility to withdraw, and processing of personal data. In addition, they had the opportunity to contact the researcher for additional information [46]. Each subject gave informed consent to participate in the study.

### Trustworthiness of the Study

The trustworthiness was assessed from the perspectives of credibility, confirmability, transferability, and dependability [47]. To increase credibility, OHNs who manage and analyze work ability risks in their work and who had experience in the subject were selected to be interviewed. Further, to ensure credibility, the data analysis was described in detail. In the analysis phase, theoretical saturation [43] was reached [39]. The confirmability of the study was enhanced by giving accurate descriptions and returning to the original data several times during the analysis. Transferability of the results is enabled by a detailed description of the context, sample, and methods [48]. The primary author, who collected and analyzed the data, has a background in occupational health care, which helped in understanding the attitudes and experiences of OHNs regarding the management and analysis of work ability risks. This may have contributed to the reflective nature of the research. The confirmability of the research is increased by the fact that the progress of the research is accurately described. The SRQR checklist, which stands for qualitative research reporting standards, was used in writing the research article [49].

## Conclusion

The participants described the management and analysis of work ability risks in diverse ways and approached these processes in a parallel manner. The management and analysis of work ability risks were perceived to be central core work in occupational health care and emphasized the importance of understanding individual-level and organizational-level WARMA. The use of electronic tools, sufficient time resources, occupational health cooperation, multi-professional cooperation, and work experience were considered to be promoting factors. On the other hand, insufficient time resources and productivity pressures were identified as hindering factors for management and analysis of work ability risks. This study provided new information that can be used to improve work ability risk management processes in occupational health care. The results of this study provide a foundation for further research, which could focus on assessing the overall competence of OHNs in WARMA and on enhancing and evaluating their competence by improving occupational health nurse training. Effective management and analysis of work ability risks are likely to have positive financial implications for customer companies, among others, but this assertion requires further research. Research can help identify and improve occupational health care training, continuing education programs, and occupational health care practices.

## Data Availability

Data are provided within the manuscript.
