# Correction: Occupational Health Nurses’ Perceptions in Work Ability Risk Management and Analysis

**DOI:** 10.1007/s10926-025-10292-5

**Published:** 2025-04-07

**Authors:** Johanna Sirkka, Riitta Suhonen, Juha Liira, Minna Stolt

**Affiliations:** 1 Department of Nursing Science, University of Turku, Turku, Finland; 2 Department of Nursing Science and Director of Nursing, University of Turku, Turku University Hospital, the Wellbeing Services County of Southwest Finland, Turku, Finland; 3 Clinical Department, University of Turku, Turku, Finland; 4 Department of Nursing Science, University of Turku, Turku, Finland; 5 The Wellbeing Services County of Satakunta, Pori, Finland

**Correction to: Journal of Occupational Rehabilitation** 10.1007/s10926-025-10282-7

In the original version of this article, the given and family names of all the authors ‘Johanna Sirkka, Riitta Suhonen, Juha Liira, Minna Stolt’ were incorrectly structured. The names were displayed correctly in all versions at the time of publication.

The affiliation details for the author Minna Stolt were incorrectly given as ‘Department of Nursing Science and the Wellbeing Services County of Satakunta, University of Turku, Pori, Finland’ but should have been ‘University of Turku, Department of Nursing Science, Turku, Finland, and the Wellbeing Services County of Satakunta, Pori, Finland’.

The original article has been corrected.

